# Cancer diagnosis and suicide outcomes: prevalence and risk meta-analysis

**DOI:** 10.1192/j.eurpsy.2022.2164

**Published:** 2022-09-01

**Authors:** G. Peviani, D. Casu, W. Mansi, M. De Prisco, F. Madeddu, J. López-Castroman, M. Fornaro, R. Calati

**Affiliations:** 1 University of Milano-Bicocca , Department Of Psychology, Milan, Italy; 2 University School of Medicine Federico II, Section Of Psychiatry - Department Of Neuroscience, Reproductive Sciences And Dentistry, Naples, Italy; 3 Nimes University Hospital, Psychiatry, Nîmes, France

**Keywords:** Suicide outcomes, Suicidology, cancer, meta-analysis

## Abstract

**Introduction:**

Available meta-analytic evidence suggests an increased risk of suicide among cancer patients, although most of the reports focused on the sole suicide death (SD) outcome and they are usually hampered by significant between-study heterogeneity.

**Objectives:**

The present meta-analysis aimed at assessing the prevalence and risk rates of SD, suicide attempt (SA), and suicidal ideation (SI) among cancer patients.

**Methods:**

Systematic search up to April 2021 of observational studies documenting cancer and suicide outcomes associations. Pooled prevalence estimates, odd ratios (ORs), risk ratios (RRs), and hazard ratios (HRs) of SD, SA, and SI were computed according to the random-effects model. SD prevalence underwent cumulative and sub-group analyses for different variables. Risk estimates underwent sensitivity analysis for study design.

**Results:**

Overall, thirty-nine studies were included. A higher risk of SD based on HR, SA based on OR and HR, and SI based on each measure was recorded among cancer patients versus controls. OR and RR of SD were not significant. Pooled prevalence rates of SD, SA and SI among cancer patients were 1.9% (1.1-3.1%), 1.4% (0.3-7.1%), and 9.1% (5.8-14.0%), respectively. Although high between-study heterogeneity held upon sensitivity and sub-group analyses, the overall message brought by risk analyses likewise held true. Age, country, study design, cancer type, sample size, cases type and comparison affected SD prevalence estimates in cancer patients. SD prevalence decreased over time.

**Conclusions:**

Cancer patients face higher risk for SA and SI versus controls. SD’ results were controversial. Cancer patients have higher prevalence rates of suicide outcomes compared to the general population.

**Disclosure:**

No significant relationships.

